# Unintended pregnancy and maternal health complications: cross-sectional analysis of data from rural Uttar Pradesh, India

**DOI:** 10.1186/s12884-020-2848-8

**Published:** 2020-03-30

**Authors:** Nabamallika Dehingia, Anvita Dixit, Yamini Atmavilas, Dharmendra Chandurkar, Kultar Singh, Jay Silverman, Anita Raj

**Affiliations:** 1 Center on Gender Equity and Health, Division of Global Public Health, University of California, San Diego School of Medicine, La Jolla, CA USA; 2 Joint Doctoral Program in Public Health (Global Health track), University of California San Diego/San Diego State University, San Diego, USA; 3 Bill and Melinda Gates Foundation, 5th Floor, Capital Court, Olof Palme Marg, Munirka, New Delhi, India; 4 Sambodhi Research and Communications Pvt. Ltd., C-126, Sector -2, Noida, Uttar Pradesh 201301 India

**Keywords:** Maternal health, Unintended pregnancy, Maternal complications, Pregnancy care, India

## Abstract

**Background:**

This study aims to explore the potential association between unintended pregnancy and maternal health complications. Secondarily, we test whether antenatal care (ANC) and community health worker (CHW) visits moderate the observed association between unintended pregnancy and maternal health complications.

**Methods:**

Cross sectional data were collected using a multistage sampling design to identify women who had a live birth in the last 12 months across 25 highest risk districts of Uttar Pradesh (*N* = 3659). Participants were surveyed on demographics, unintendedness of last pregnancy, receipt of ANC clinical visits and community outreach during pregnancy, and maternal complications. Regression models described the relations between unintended pregnancy and maternal complications. To determine if receipt of ANC and CHW visits in pregnancy moderated associations between unintended pregnancy and maternal complications, we used the Mantel-Haenzel risk estimation test and stratified logistic models testing interactions of unintended pregnancy and receipt of health services to predict maternal complications.

**Results:**

Around one-fifth of the women (16.9%) reported that their previous pregnancy was unintended. Logistic regression analyses revealed that unintended pregnancy was significantly associated with maternal complications- pre-eclampsia (AOR:2.06; 95% CI:1.57–2.72), postpartum hemorrhage (AOR:1.46; 95% CI: 1.01–2.13) and postpartum pre-eclampsia (AOR:2.34; 95% CI:1.47–3.72). Results from the Mantel Haenszel test indicated that both ANC and CHW home visit in pregnancy significantly affect the association between unintended pregnancy and postpartum hemorrhage (*p* < 0.001).

**Conclusion:**

Unintended pregnancy is associated with increased risk for maternal health complications, but provision of ANC clinical visits and CHW home visits in pregnancy may be able to reduce potential effects of unintended pregnancy on maternal health.

## Background

Each year, approximately 85 million women in the world face an unintended pregnancy [1], more than one in seven of these cases occurs in India [2]. Global evidence, including that from higher income settings, suggests that unintended pregnancy increases risk for maternal morbidity and mortality [3, 4]. Studies from India indicate that unintended pregnancy is associated with lower maternal health care utilization and poorer infant health outcomes [5, 6]. However, research on associations of unintended pregnancy and maternal health in India remain lacking. This study assesses unintended pregnancy and its association with maternal health complications among women in Uttar Pradesh, India.

Uttar Pradesh, the most populous state in India (population of approximately 200 million) has twice the unwanted fertility rate as that seen for India as a whole (1.65 vs. 0.80) [7], and a much higher maternal mortality rate as well (285 maternal deaths per 100,000 live births vs. 167 per 100,000 live births in India) [8]. Heightened maternal risk in Uttar Pradesh can be attributed, at least in part, to poorer maternal health care utilization, including antenatal care (ANC) [9]. Studies from India and elsewhere indicate that women with an unintended pregnancy are less likely to recieve ANC [6, 10–16]. In India, ANC care has received expanded attention via home visits to pregnant women by community outreach workers known as Accredited Social Health Activists- ASHAs, with a focus on socially vulnerable women (e.g., poorer, less educated, adolescents); although vulnerable women are more likely to be approached via ASHAs relative to clinicians, not all women are reached [17]. Given the heightened risk for unintended pregnancy and related complications among socially vulnerable women [18, 19], ASHA contacts during pregnancy may be particularly important, but these have received little focus in the research literature.

This study examines unintended pregnancy among women in Uttar Pradesh and assesses the association between unintended pregnancy and three key maternal health complications,pre-eclampsia, postpartum hemorrhage and postpartum pre-eclampsia. Secondarily, the study explores whether ANC moderates the observed associations between unintended pregnancy and maternal health complications, considering both ANC from a clinician and ASHA home visits. These findings may offer important insight into how to reduce the potential effects of an unintended pregnancy on maternal health in India.

## Methods

This study involved cross-sectional analysis of household survey data collected from women who delivered a live birth in the past 12 months. Data were collected as part of an evaluation study of a public health system strengthening intervention in Uttar Pradesh. This intervention, known as the Technical Support Unit (TSU), was not focused specifically on family planning or unintended pregnancy, but designed to support maternal and child health care utilization. Data from the second cross-sectional survey of evaluation was used for this analysis.

These data were collected from 100 blocks (geographic areas including a population of approximately 100,000) of the 25 lowest-performing districts of Uttar Pradesh, as identified by the Government of India and the Government of Uttar Pradesh based on the districts’ maternal mortality ratios, percentage of facility deliveries, infant mortality rates, percentage of children 12–23 months fully immunized, total fertility rates, and modern contraceptive prevalence rates. Six ASHA areas (the geographic catchment area of an ASHA) were randomly selected from within each of the 100 TSU blocks, and a census of all the households within those blocks was conducted to identify women aged 15–49 years who had a live birth in last 12 months. Data were collected from June to September 2016 from these 600 ASHA areas, and 4200 women meeting inclusion criteria were randomly sampled. Of these, 3703 consented (% of recruited) and were interviewed. Our analyses were restricted to the subsample of non-pregnant women (*n* = 3659), as pregnancy intent of prior birth was not assessed for those women who were currently pregnant.

Women were interviewed by trained female staff using mobile tablets. Interviewers were trained on accurate and ethical collection of demographic and maternal health related information. Written and informed consent was obtained for every woman that agreed to participate. The interviewers explained the purpose of the study to the mothers in their local dialect and asked them whether they were willing to participate. Adolescent mothers younger than 18 years of age provided assent while a guardian provided formal verbal informed consent. Study protocols were reviewed and approved by the PHS-ERB (an independent ethical review board) and the Health Ministry Screening Committee’s (HMSC) Indian Council for Medical Research (ICMR). These protocols were also registered with the Clinical Trial Registry – India (CTRI/2015/09/006219).

### Measures

The dependent variables in this analysis were self-reported responses for three complications - pre-eclampsia during pregnancy, postpartum hemorrhage and postpartum pre-eclampsia. All of these were specific to the index pregnancy delivered in the past year. Following the WHO [20] and National Institute for Health and Care Excellence guidelines [21] and National Family Health Survey (NFHS) coding by Agarwal et al. [22], we created a dichotomous indicator for pre-eclampsia; women who reported both difficulty in vision and swelling of the legs, body or face were coded as having symptoms suggestive of pre-eclampsia. Postpartum hemorrhage included women who reported both excessive bleeding and difficulty in breathing after delivery, and postpartum pre-eclampsia included severe headache and difficulty in vision after delivery. These two indicators were constructed based on WHO [20] and ACOG [23] guidelines. The three specific complications were chosen for analysis because of availability of data on their respective symptoms and these complications being critical to the burden of maternal deaths in India [24, 25]. Additionally, we also assessed experience of any complications during pregnancy, childbirth and postpartum, based on self-reported symptoms experienced by women during the respective timeframes. *Pregnancy complications* assessed included excessive vaginal bleeding, high blood pressure, swelling of hands and feet, headaches, convulsions, severe abdominal pain which is not labour pain, breathing difficulty, high fever, loss of consciousness and blurred vision. *Delivery complications* were defined as premature labour, premature rupture of membrane, retained placenta, obstructed labour and breech/mal presentation. *Postpartum complications* included excessive bleeding after delivery, high blood pressure, convulsions, foul smelling vaginal discharge, fever, severe headache, loss of consciousness and blurred vision, during the first month after delivery. The list of these items was developed based on National Family Health Survey (NFHS)-3 [7], and formal discussions with maternal health experts during the development of the survey. Each symptom was asked as a yes no item. Each of the complications variables were dichotomized as either yes for any symptom or no on all assessed symptoms for that timeframe.

The independent variable, *unintended pregnancy*, was also taken from the NFHS [7] and asked women to recall their feelings at the time they became pregnant in their index pregnancy-- “At the time you became pregnant, did you want to become pregnant then, did you want to wait until later, or did you not want to have any/any more children at all?” “Later” and “Not at all” responses were collapsed together to indicate unintended pregnancy.

Covariates considered as potential confounders included receipt of *minimum 4 ANC visits (based on current World Health Organization (WHO) standard for adequate ANC)* [26] and receipt of any *home visit by ASHA* during pregnancy. Women who had at least 4 ANC visits during their recent pregnancy, either at home or at a health facility, wherein any of the WHO recommended ANC tests (weight, blood pressure, abdomen, ultrasound, haemoglobin, urine etc.) were conducted were labelled as having received minimum 4 ANC visits. Receipt of any home visit by ASHA was measured via a single dichotomous variable that assessed whether a woman had received any home visit from an ASHA during her recent pregnancy.

Additional covariates included socio-demographic and index child-related variables, intimate partner violence (IPV), and institutional delivery. Sociodemographic items on the woman and her husband included maternal age, maternal age at marriage (categorized as < 18 or ≥ 18), maternal and husband’s education (each categorized as none, 1–5 yrs., 6–10 yrs., more than 10 yrs), caste (categorized as Scheduled Caste/Scheduled Tribe- SC/ST, Other Backward Caste- OBC, and General Caste), religion (dichotomized as Muslim and non-Muslim), and household wealth. The Standard of Living Index (SLI) was used as a proxy indicator for characterizing household wealth; the SLI methodology is used for this purpose in the Demographic and Health Surveys across multiple national contexts, including India [27]. SLI scores were categorized into quartiles based on scores of 0–33, 33–66, and 66–100 (range 0–100) to create low, medium and high household wealth categories. Covariates related to the fertility or the index child included parity (categorized as 1, 2, 3+ births) and singleton vs. multiple birth,time since birth (categorized as ≤1 mo, 2–6 mo, > 6 mo).

IPV was measured via 12 questions related to women’s experiences of physical or sexual violence from their current husband in their lifetime, taken from the NFHS [7]. Women reporting yes on any of the 12 items were identified as having experienced IPV. Institutional delivery was assessed via an item on whether they delivered at any government health facility, privately owned hospital/clinic or an NGO hospital/clinic for the index childbirth. As the survey did not reliably obtain information on the qualifications of the provider at delivery, skilled birth attendant could not be included.

### Statistical analysis

Descriptive analyses were used to characterize the sample, as a whole and by whether their index pregnancy was unintended or intended. Chi square analyses were used to assess associations of all covariates with unintended pregnancy, and then with each maternal health outcome. Unadjusted logistic regression models were used to determine whether unintended pregnancy was independently associated with each maternal complication. Next, three separate adjusted logistic regression models assessed the relationship between unintended pregnancy and the three maternal complications – pre-eclampsia, postpartum hemorrhage and postpartum pre-eclampsia. To adopt a parsimonious model while assessing the relationship between unintended pregnancy and maternal complications, the variables with established relevance [28–30]– maternal age, maternal education, wealth index, and variables with *p* value less than 0.25 for unadjusted odds were considered as covariates [31].

To understand interaction effects of unintended pregnancy and pregnancy care, a multiplicative statistical interaction term comprising unintended pregnancy and each of the two variables of pregnancy care - receipt of minimum 4 ANC and receipt of home visit by ASHA during pregnancy - was entered into a model accounting for all the covariates included in the logistic regression models, to identify their effect on each maternal complication indicator. Wald tests were used to assess the significance of interaction terms - ANC x unintended pregnancy and ASHA visit x unintended pregnancy - for each of the complications outcomes; interactions were deemed significant at the level of *p* < 0.05. Additionally, the Mantel-Haenzel risk estimation test was also used to determine if the interaction of the confounders with unintended pregnancies leads to significant change in the risk of occurrence of complications. The nature of significant interactions was further assessed using stratified logistic regression models.

Sample weights calculated based on the multistage sampling design were utilized in all analyses. Data were analyzed using STATA 13.0 software (StataCorp, USA).

### Results

Participants (*n* = 3659) were aged 16–49 years (mean age = 26, std. dev = 4.4). Half (48.1%) have no formal education. Almost one-third (29.8%) were married as minors, and 44.3% report IPV from their current husband; 44.9% had three or more children. Minimum 4 ANC at index pregnancy was reported by 12.4%, and no ASHA home visit in that pregnancy was reported by 39.7%; 30.7% reported that their index child was delivered at home. Chi-square analyses indicated that women reporting marriage as a minor, no education, and lowest wealth were more likely to report an unintended pregnancy, as were those with higher parity, a multiparous birth, and no or less than 4 ANC.

Around 16% of women reported pre-eclampsia during their most recent pregnancy, 7.0% reported postpartum hemorrhage and 5.4% reported to have experienced postpartum pre-eclampsia. No statistically significant differences were observed for complications between institutional and home deliveries. Chi-square analyses indicated significant associations between unintended pregnancies and each maternal complication. Having less than 4 ANC and no ASHA home visit in pregnancy were also significantly associated with pre-eclampsia, postpartum hemorrhage and postpartum pre-eclampsia. IPV was also significantly associated with pre-eclampsia. No other covariates were significantly associated with the outcomes. (See Table 1).

**Table 1:** Sample characteristics for total sample by pregnancy intent, experiences of pre-eclampsia, postpartum hemorrhage and postpartum pre-eclampsia, respectively among women in Uttar Pradesh who gave birth in the past year (*N* = 3659)

		Total sample	Unintended pregnancy		Pre-eclampsia		Postpartum hemorrhage		Postpartum pre-eclampsia	
		(Unwtd N = 3659)	(Unwtd *N* = 619)		(Unwtd *N* = 583)		(Unwtd *N* = 257)		(Unwtd *N* = 198)	
		Wtd%/Mean(SD)	Wtd%/Mean (SD)	p value*	Wtd%/Mean(SD)	p value*	Wtd%/Mean(SD)	p value*	Wtd%/Mean(SD)	p value*
		(n)^a^	(n)^a^		(n)^a^		(n)^a^		(n)^a^	
Maternal age		26.4(4.4)	27.6(4.9)	0.00	26.5 (4.4)	0.70	25.9 (4.1)	0.11	26.4 (4.0)	0.89
Maternal age at marriage	Less than 18 years	29.8	23.1	0.00	14.9	0.18	6.5	0.14	5.8	0.68
		1092	240		163		72		55	
	Equal or more than 18 years	70.2	15.4		17.1		8.2		5.4	
		2567	379		420		185		143	
Maternal education	Never attended school	48.1	20.4	0.00	16.4	0.69	7.3	0.20	5.2	0.90
		1741	337		281		111		88	
	1 5 years in school	15.2	15.1		15.2		6.8		5.5	
		544	84		84		39		29	
	6 9 years in school	15.2	13.2		15.9		7.0		5.8	
		575	71		90		44		40	
	> = 10 years in school	21.5	16.8		18.0		9.8		6.0	
		799	126		128		63		41	
Husband’s education	Never attended school	24.8	18.6	0.24	15.6	0.78	5.9	0.13	5	0.71
		856	151		133		47		50	
	1 5 years in school	13.8	21.1		16.6		6.4		4.2	
		515	90		80		31		21	
	6 9 years in school	27.5	16.9		16.1		7.8		5.8	
		1013	176		169		77		54	
	> = 10 years in school	33.9	16.4		17.5		9.5		6.1	
		1275	201		200		101		72	
Caste	SC/ST	29.3	20.1	0.17	17.2	0.18	6.6	0.48	5.1	0.35
		1042	207		178		70		60	
	OBC	54.9	16.5		15.4		8.1		5.2	
		2021	320		303		145		101	
	General	15.8	17.4		19.1		8.2		7.0	
		596	92		102		42		37	
Religion	Muslim	17.2	18.9	0.49			7.6	0.68	5.4	0.48
		610	103				212		163	
	Non muslim	82.8	17.5				8.2		6.1	
		3049	516				45		35	
Wealth index	Low	63.5	19.3	0.04	16.4	0.95	7.6	0.93	4.9	0.28
		2261	433		364		161		116	
	Medium	27.8	15.4		16.4		8		7.0	
		1056	144		167		77		70	
	High	8.7	13.7		17.2		7.4		5.3	
		342	42		52		19		12	
Birth parity	1	28.2	11.3	0.00	17.5	0.44	8.0	0.66	5.9	0.83
		1061	117		161		76		57	
	2	26.1	16.5		15.0		8.3		5.5	
		956	131		141		68		51	
	3+	44.9	22.2		16.7		7.2		5.2	
		1641	370		281		113		90	
Singleton birth	Singleton	99.3	17.6	0.02	16.5	0.43	7.7	0.25	5.4	0.25
		3630	607		581		255		195	
	Multiple births	0.7	38.9		9.4		15.4		17.7	
		29	12		2		2		3	
Time since birth	Less than or equal to 2 months	24.9	18.3	0.71	17.9	0.17	7.7	0.66	5.8	0.32
		889	153		153		54		47	
	More than 2 months and less than 7 months	31.9	18.2		17.4		8.4		6.3	
		1190	200		188		92		74	
	More than 6 months	43.2	17		15.0		7.2		4.8	
		1580	266		242		111		77	
Intimate partner violence(IPV)	IPV ever	56.4	16.6	0.57	19.9	0.00	8.5	0.22	6.1	0.19
		2093	811		354		145		109	
	No IPV ever	43.6	18.1		12.9		8.9		4.9	
		1566	357		229		112		89	
Institutional delivery	Facility delivery	69.3	17.3	0.24	16.0	0.40	7.6	0.68	5.2	0.29
		2569	262		395		183		140	
	Delivery at home	30.7	19.2		17.5		8.1		6.2	
		1090	206		188		74		58	
Receipt of minimum four ANC	Received minimum four ANC	12.4	11.7	0	11.6	0.01	4.7	0.02	5.8	0.81
		473	51		52		23		30	
	Not received at least four ANC	87.6	18.6		17.2		8.1		5.5	
		3186	568		100		234		168	
Receipt of home visit by ASHA	Received at least one home visit by ASHA	60.3	16.5	0.06	14.0	0.00	6.5	0.01	4.6	0.03
		2241	348		313		132		107	
	Not received at least one home visit by ASHA	39.7	19.6		20.2		9.5		6.9	
		1418	271		270		125		91	
Unintended pregnancy	Intended pregnancy	–	–	–	14.4	0.00	7.2	0.03	4.6	0.00
					437		203		149	
	Unintended pregnancy	–	–		26.3		10.3		9.8	
					146		54		49	

^a^Results presented are weighted to account for sampling design

*p values assess differences between groups who had an unintended pregnancy and an intended pregnancy, based on chi square analyses for categorical variables and t-tests for continuous variables

Both unadjusted and adjusted logistic regression analyses revealed that unintended pregnancy was significantly associated with pre-eclampsia (AOR:2.06; 95% CI:1.57–2.75), postpartum hemorrhage (AOR:1.46; 95% CI: 1.01–2.13) and postpartum pre-eclampsia (AOR:2.34; 95% CI: 1.47–3.72). In terms of the potential confounders, participants reporting no or less than 4 ANC were significantly more likely to report pre-eclampsia (AOR: 1.45; 95% CI: 1.004–2.09) and postpartum hemorrhage (AOR: 1.77; 95% CI: 1.06–2.97), but not postpartum pre-eclampsia. Those reporting no ASHA visit during pregnancy were more likely to report all three complications (AOR: 1.49; 95% CI:1.15–1.93 for pre-eclampsia, AOR: 1.48; 95% CI:1.06–2.06 for postpartum hemorrhage and AOR: 1.52; 95% CI:1.02–2.25 for postpartum pre-eclampsia). Additional variables associated with pre-eclampsia included IPV. No other variables were significantly associated with the complications outcomes. (See Table 2).

**Table 2:** Multivariate logistic regression of factors associated with experiences of pre-eclampsia, postpartum hemorrhage and postpartum pre-eclampsia, respectively, among women in Uttar Pradesh who gave birth in the past year (N = 3659)

		Pre-eclampsia^+^		Postpartum hemorrhage+		Postpartum pre-eclampsia	
		Crude OR (95% CI)	Adjusted OR (95% CI)	Crude OR (95% CI)	Adjusted OR (95% CI)	Crude OR (95% CI)	Adjusted OR (95% CI)
Unintended pregnancy	Intended/planned pregnancy	Ref	Ref	Ref	Ref	Ref	Ref
	Unintended pregnancy	2.12(1.62–2.75) ***	2.06 (1.57–2.72) ***	1.49 (1.02–2.17) **	1.46 (1.01–2.13) **	2.29 (1.45–3.61) ***	2.34 (1.47–3.72) ***
Receipt of at least four ANC	Received at least four ANC	Ref	Ref	Ref	Ref	Ref	Ref
	Not received at least four ANC	1.57 (1.11–2.24) ***	1.45 (1.004–2.09) **	1.81 (1.05–3.08) **	1.77 (1.06–2.97) **	0.94 (0.59–1.50)	0.87 (0.53–1.43)
Receipt of home visit by ASHA	Received at least one home visit by ASHA	Ref	Ref	Ref	Ref	Ref	Ref
	Not received any home visit by ASHA	1.56 (1.20–2.01) ***	1.49 (1.15–1.93) **	1.51 (1.08–2.09) **	1.48 (1.06–2.06) **	1.52 (1.02–2.29) **	1.52 (1.02–2.25) **
Institutional delivery	Facility	Ref	–	Ref	–	Ref	–
	Home	1.11 (0.86–1.42)	–	1.07 (0.76–1.53)	–	1.22 (0.84–1.76)	–
IPV	Experienced IPV in lifetime	Ref	Ref	Ref	Ref	Ref	Ref
	Experienced no IPV in lifetime	0.59 (0.45–0.77) ***	0.60 (0.46–0.78) ***	0.78 (0.53–1.15)	–	0.79 (0.55–1.12)	–
Maternal age	Continuous	Ref	Ref	Ref	Ref	Ref	Ref
		1.004 (0.98–1.03)	0.99 (0.97–1.02)	0.97 (0.94–1.01)	0.97 (0.93–1.01)	1.002 (0.96–1.04)	0.99 (0.96–1.04)
Maternal age at marriage	Less than 18 years	Ref	–	Ref	–	Ref	–
	Equal to or more than 18 years	1.17 (0.92–1.49)	–	1.29 (0.91–1.82)	–	0.92 (0.63–1.34)	–
Maternal education	> = 10 years in school	Ref	Ref	Ref	Ref	Ref	Ref
	6–9 years in school	0.92 (0.69–1.21)	0.85 (0.57–1.25)	0.70 (0.45–1.07)	0.65 (0.43–0.99) **	0.96 (0.53–1.74)	1.03 (0.58–1.82)
	1–5 years in school	0.97 (0.68–1.36)	0.76 (0.50–1.15)	0.67 (0.40–1.12)	0.63 (0.38–1.05)	0.90 (0.46–1.73)	0.95 (0.50–1.81)
	Never attended school	1.12 (0.83–1.54)	0.81 (0.57–1.15)	0.72 (0.47–1.11)	0.67 (0.43–1.05)	0.85 (0.49–1.47)	0.88 (0.52–1.51)
Husband’s education	> = 10 years in school	Ref	–	Ref	–	Ref	–
	6–9 years in school	1.07 (0.73–1.57)	–	1.09 (0.57–2.07)	–	0.83 (0.35–1.96)	–
	1–5 years in school	1.04 (0.73–1.47)	–	1.36 (0.88–2.08)	–	1.16 (0.73–1.83)	–
	Never attended school	1.15 (0.83–1.59)	–	1.67 (1.07–2.60)	–	1.22 (0.72–2.07)	–
Caste	General	Ref	–	Ref	–	Ref	–
	OBC	0.87 (0.67–1.12)	–	1.24 (0.84–1.83)	–	1.02 (0.64–1.64)	–
	SC/ST	1.13 (0.84–1.52)	–	1.25 (0.80–1.97)	–	1.39 (0.89–2.18)	–
Religion	Non-muslim	Ref	Ref	Ref	Ref	Ref	Ref
	Muslim	1.12 (0.82–1.54)	1.12 (0.82–1.53)	1.09 (0.71–1.67)	1.15 (0.75–1.75)	1.14 (0.78–1.69)	1.14 (0.76–1.71)
Wealth index	High	Ref	Ref	Ref	Ref	Ref	Ref
	Medium	0.99 (0.76–1.29)	0.99 (0.66–1.49)	1.09 (0.56–2.11)	1.22 (0.65–2.30)	1.35 (0.50–3.59)	1.40 (0.56–3.53)
	Low	1.06 (0.72–1.55)	1.02 (0.68–1.52)	1.03 (0.55–1.92)	1.24 (0.69–2.22)	0.91 (0.37–2.24)	0.94 (0.40–2.21)
Birth parity	1	Ref	–	Ref	–	Ref	–
	2	0.83 (0.63–1.08)	–	1.04 (0.70–1.53)	–	0.92 (0.52–1.63)	–
	3+	0.94 (0.73–1.21)	–	0.89 (0.65–1.22)	–	0.87 (0.56–1.35)	–
Singleton Birth	Singleton	Ref	Ref	Ref	Ref	Ref	Ref
	Multiple births	0.52 (0.09–1.75)	–	2.19 (0.54–8.83)	1.81 (0.48–6.81)	3.75 (1.09–12.89)	–
Time since birth	Less than or equal to 2 months	Ref	–	Ref	–	Ref	–
	More than 2 months and less than 7 months	0.96 (0.75–1.22)	–	1.09 (0.67–1.75)	–	1.08 (0.67–1.73)	–
	More than 6 months	0.81 (0.64–1.01)	–	0.93 (0.62–1.38)	–	0.81 (0.54–1.21)	–

**Statistically significant at *p* < 0.05

***Statistically significant at *p* < 0.01

Results presented in Table 2 are weighted to account for sampling design

Interaction terms were significant for postpartum hemorrhage, but not for pre-eclampsia and postpartum pre-eclampsia. Results from the Mantel Haenszel test indicate that both ANC and ASHA home visit significantly affect the association between unintended pregnancy and postpartum hemorrhage (*p* < 0.001). Among women who did not recieve at least 4 ANC visits (as compared to women who had 4 or more visits), those with an unintended pregnancy were at higher odds of experiencing postpartum hemorrhage. Similar results were observed after stratification by ASHA visits. (See Table 3).

**Table 3:** Association between unintended pregnancy and experience of pre-eclampsia and postpartum haemorrhage, stratified by minimum 4 ANC and ASHA home visit (N = 3659)

		Postpartum hemorrhage
		Adjusted OR (95% CI) ^**++**^
Not received minimum 4 ANC	Intended pregnancy	Ref
	Unintended pregnancy	1.57 (1.09–2.28) **
Not received any home visit by ASHA	Intended pregnancy	Ref
	Unintended pregnancy	2.83 (1.77–4.51) ***

**Statistically significant at *p* < 0.05

***Statistically significant at *p* < 0.01

^**+**^Covariates include IPV, maternal age, maternal education, wealth and religion ++ Covariates include maternal age, maternal education, wealth, religion and singleton birth

Results presented in Table 3 are weighted to account for sampling design

Three separate models for complications during pregnancy, childbirth and postpartum were also assessed. Unintended pregnancy was observed to be significantly associated with complications during pregnancy (AOR: 1.67; 95% CI: 1.34–2.08, delivery (AOR: 1.29; 95% CI: 1.03–1.63), and postpartum (AOR: 1.32; 95% CI: 1.07–1.61), even after adjusting for all covariates. (Supplement table).

## Discussion

Current findings suggest that one in every five pregnancies in the 25 high priority districts of Uttar Pradesh is unintended. Although lower than the estimates for the state (34.7%), it is far higher than desirable, especially since these distrcits bear the maximum burden of maternal mortality within Uttar Pradesh. In accordance with prior research [4, 32], this study found that an unintended pregnancy is associated with pre-eclampsia, postpartum haemorrhage and postpartum pre-eclampsia, which may contribute to the high maternal mortality rates in the region [33, 34]. Study findings reinforce the important role of family planning interventions in improving maternal and child health. Studies have indicated the association between contraceptive use and maternal deaths [35, 36] as well as child health [37, 38]; unintended pregnancy could be the plausible mediator for this relationship. With access to reproductive health care, women can avoid unintended pregnancies and consequently, complications during pregnancy and childbirth.

While many studies have documented this association between unintended pregnancies and maternal complications, they have not explored the effect of care during pregnancy on this relationship. In this study we also considered the inter-relationship between unintended pregnancy, maternal health care, and maternal complications. Consistent with prior research [5, 12, 16], we found that women with unintended pregnancies were less likely to receive adequate ANC, and, also as seen in prior research [39, 40], we found that non-receipt of adequate ANC or home visits by an ASHA during pregnancy were associated with increased risk for complications. Extending upon this, we found a significant interaction between unintended pregnancy and non-receipt of ANC or ASHA care, such that the combination of these placed women at greatest risk for postpartum hemorrhage. The reasons why women with unintended pregnancies receive insufficient care during pregnancy are unclear. One possibility is that women with unintended pregnancies are likely to recognize their pregnancy in a later stage, which means they miss the opportunity to access comprehensive maternal health services during pregnancy [41]. Another explanation could be that women with an unintended pregnancy may be less pro-active about seeking out health care [14]. Notably, the association between unintended pregnancy and maternal complications holds true even after accounting for health service utilization (ANC and ASHA home visits) during pregnancy. Factors such as stress associated with pregnancy could possibly explain this relationship. Further qualitative research may be useful to elucidate mechanisms of the observed association.

The observed interaction also suggests that for women with an unintended pregnancy, receipt of ANC or antenatal ASHA home visits may attenuate potential effects of unintended pregnancy on complications in pregnancy. Women more vulnerable to unintended pregnancy, such as those who are poorer, less educated, and married as minors [42, 43], as well as those who have experienced IPV [19], may most benefit from ANC and ASHA visits and should be prioritized for these.

As noted above, effects of ANC was not observed for postpartum pre-eclampsia. Complications post childbirth may be associated with other factors not included in our model, such as provision of care during delivery (eg., active management of third stage of labor) [44, 45]. Provision of care during the post-natal period is equally critical, in terms of preventing postpartum complications [46]. Continuum of care throughout pregnancy, birth and after delivery are key to preventing life-threatening complications during the three critical stages of maternal health [47, 48], regardless of pregnancy intendedness. Certainly, an element of this must include contraceptive use, as this is key to averting unintended pregnancies and resultant complications.

### Limitations

Although the current study provides important insights into the effect of unintended pregnancy on maternal complications, there are a few limitations to the study. First being experience of complications influencing intendedness, which may explain the observed association between unintended pregnancy and complications. Another key concern is the reliability of self-reported measures of unintended pregnancies and complications. Literature suggests that women tend to rationalize unwanted births as wanted, post the birth of the child [49]. Retrospective reporting of pregnancy desire may be influenced by the experience of parenthood post the birth of the child. Studies conducted in developed as well as developing countries show that the actual proportion of unintended pregnancies is much higher than the estimates from retrospective reporting [50, 51]. A longitudinal study conducted in India found the actual percentage of unintended pregnancies to be at least 15 percentage points higher than retrospectively reported estimates, across four states in the country [49]. For information related to complications, clinical data was unavailable to validate the self-reported responses. Our sample included women who gave birth in health facilities, as well as at home. It is possible that the reported prevalence of complications is smaller than the actual prevalence, among women who gave birth at home, owing to social acceptability bias. No study to our knowledge, has examined this differential effect of place of delivery on reported estimates. Although, studies have indicated absence of significant inconsistencies between reporting of maternal health complications in 1 week vs 6 months, by place of delivery [52]. Future studies are required to examine these relationships in the Indian context. Recall bias could have also altered responses regarding both pregnancy intentions and complications, based on increasing time since birth of the child. Third, the survey did not capture information related to contraceptive use prior to the pregnancy. This would be key to understanding contribution of family planning towards maternal complications. Fourth, because this is a cross-sectional study, inferences cannot readily be made about causality. Finally, the findings are applicable to the 25 HPDs of UP, and are not generalizable to all of UP or other states of India. However, they do offer some insights into situations in similar settings with disproportionate burden of unintended pregnancies.

## Conclusions

The current study documents the association between unintended pregnancies and pregnancy complications, which in turn are associated with complications in pregnancy and postpartum; further analysis also revealed that the association was mitigated by receipt of care during pregnancy. Findings highlight the need for a focus on a continuum of care for improved maternal health. Within the continuum, women should have access to reproductive health choices, including the choice to get prengnant or not. This study suggests that the exercise of choice may have a beneficial effect on maternal health. Even if choices are constrained, our findings indicate that the heatlh care provision during pregnancy may play a crucial role in reducing the maternal health risks associated with unintended pregnancies.

## Data Availability

The dataset supporting the conclusions of this article is available in the Harvard Dataverse, 10.7910/DVN/LZGW76

## Abbreviations

ANC: Ante natal care
AOR: Adjusted odds ratio
ASHA: Accredited social health activist
CI: Confidence interval
HMSC: Health Ministry Screening Committee
ICMR: Indian Council for Medical Research
IPV: Intimate partner violence
IRB: Institutional review board
NFHS: National Family Health Survey
OBC: Other backward classes
PHS-ERB: Public Health service- ethical review board
SC: Scheduled caste
SLI: Standard of living index
ST: Scheduled tribe
WHO: World Health Organization
